# Diffusion tensor imaging versus intraoperative subcortical mapping for glioma resection: a systematic review and meta-analysis

**DOI:** 10.1007/s10143-023-02058-5

**Published:** 2023-06-28

**Authors:** Yiming Li, Jiahe Guo, Kai Zhang, Huijie Wei, Jikang Fan, Shengping Yu, Tao Li, Xuejun Yang

**Affiliations:** 1 Department of Neurosurgery, Tianjin Medical University General Hospital, Tianjin, China; 2 Institute for Intelligent Healthcare, Tsinghua University, Beijing, China; 3 Department of Neurosurgery, Tsinghua University Beijing Tsinghua Changgung Hospital, Beijing, China

**Keywords:** Glioma, Diffusion tensor imaging, Intraoperative subcortical mapping, Extent of resection, Postoperative neurological deficit

## Abstract

Maintaining the integrity of crucial fiber tracts allows functional preservation and improved recovery in patients with glioma resection. Diffusion tensor imaging (DTI) and intraoperative subcortical mapping (ISM) are commonly required for pre- and intraoperative assessment of white matter fibers. This study investigated differences of clinical outcomes in glioma resection aided by DTI or ISM. A comprehensive literature retrieval of the PubMed and Embase databases identified several DTI or ISM studies in 2000–2022. Clinical data, including extent of resection (EOR) and postoperative neurological deficits, was collected and statistically analyzed. Heterogeneity was regressed by a random effect model and the Mann–Whitney U test was used to test statistical significance. Publication bias was assessed by Egger test. A total of 14 studies with a pooled cohort of 1837 patients were included. Patients undergoing DTI-navigated glioma surgery showed a higher rate of gross total resection (GTR) than ISM-assisted surgical resection (67.88%, [95% CI 0.55—0.79] vs. 45.73%, [95% CI 0.29—0.63], *P* = 0.032). The occurrence of early postoperative functional deficit (35.45%, [95% CI 0.13—0.61] vs. 35.60% [95% CI 0.20—0.53], *P* = 1.000), late postoperative functional deficit (6.00%, [95% CI 0.02—0.11] vs. 4.91% [95% CI 0.03—0.08], *P* = 1.000) and severe postoperative functional deficit (2.21%, [95% CI 0—0.08] vs. 5.93% [95% CI 0.01—0.16], *P* = 0.393) were similar between the DTI and ISM group, respectively. While DTI-navigation resulted in a higher rate of GTR, the occurrence of postoperative neurological deficits between DTI and ISM groups was comparable. Together, these data indicate that both techniques could safely facilitate glioma resection.

**Supplementary Information:**

The online version contains supplementary material available at 10.1007/s10143-023-02058-5.

## Introduction

Glioma, the most prevalent primary intracranial tumor, exhibits invasive behavior, facilitating infiltration of tumor tissue into the cerebral cortex and subcortical fiber tracts. This feature poses significant challenges to the safe and effective resection of the lesion and contributes to high mortality and morbidity [1, 2]. Neurosurgeons strive to achieve maximal safe resection during glioma surgery while balancing functional preservation with tumor removal [3, 4]. In cases where gliomas are located in eloquent areas, intraoperative brain mapping, and neuronavigation systems can significantly aid in achieving a better surgical dissection along the functional-oncological boundary [5, 6]. Intraoperative surgical mapping has been proposed as a standard of care in the neurosurgical treatment of glioma. Further, the preservation of subcortical components and protection of the cerebral cortex is crucial for ensuring a favorable functional prognosis or neural plasticity, particularly in the case of deep-seated gliomas.

Intraoperative subcortical stimulation mapping (ISM) and diffusion tensor imaging (DTI) navigation can be used in combination to better preserve subcortical structures during glioma surgery [7-9]. ISM, considered the gold standard for subcortical identification, provides superior intraoperative neurological function monitoring, which improves the extent of tumor resection and reduces the likelihood of neurological dysfunction [10-12]. Despite its superior ability to distinguish function-related fiber tracts from white matter, ISM is subject to several significant drawbacks, including patient cooperation, operator experience, and extensive prior knowledge of fiber tracts. To address these issues, DTI is often incorporated into neuronavigation systems to support preoperative assessment of the relationship between fiber tracts and the tumor and intraoperative fiber tract localization. In practice, DTI merged into a neuronavigation system can support preoperative assessment of the relationship between the fiber tracts and tumor and intraoperative localization of fiber tracts [13-15]. However, the accuracy of intraoperative fiber tract localization can be impacted by brain edema and intraoperative brain shift during neuronavigation, as well as the anatomical seed location and fractional anisotropy threshold settings [16]. Considering the benefits and limitations of each approach, advanced neurosurgery centers commonly use both techniques in combination to achieve optimal fiber tract preservation.

The widespread use of ISM is limited by its dependence on surgical experience, which has led to questions about the safety of DTI and whether it can serve as a reproducible substitute for ISM. Few studies have compared the extent of tumor resection and prognosis achieved with DTI versus ISM [17, 18]. This is the first study employs a meta-analysis methodology to examine the impact of ISM and DTI neuronavigation on the extent of lesion resection and postoperative neurological function.

## Methods

### Study design

This study was carried out according to the PRISMA guidelines for systematic reviews and meta-analysis [19-21]. Using the PICO criteria (P: patient, I: intervention, C: control, O: outcome) and PRISMA checklist [19, 20], a key question was identified: do patients suffering from glioma have a more significant percentage of gross total resection (GTR) and lower postoperative neurological deficits when receiving DTI versus ISM-assisted surgery? This study was registered to the INSPLAY registry for systematic review and meta-analysis (registration number: INPLASY202180013, https://doi.org/10.37766/inplasy2021.8.0013).

### Data sources and search strategy

The PubMed and Embase databases were accessed and used to identify relevant studies published from January 1st, 2000, to December 31st, 2022. The combination of keywords and index terms created search strategies. Three key terms were used to identify relevant studies published in the search period: “glioma”, “intraoperative subcortical mapping”, and “diffusion tensor imaging” (detailed search strategy was showed in appendix 1 and 2). In addition to those databases, additional studies were included by citation search.

### Study selection and quality assessment

Case series studies, observational studies, and randomized controlled trials (RCT) were assessed for inclusion. Included studies should meet the following criteria: (i) studies published in English; (ii) patients were pathologically diagnosed with glioma (WHO grades 1–4); (iii) DTI or ISM was used in glioma surgery; (iv) providing information on the extent of lesion resection or assessment of neurological deficits. Literatures not related to search strategy, case reports, systematic reviews, meta-analyses, studies of pediatric neurosurgery, studies of non-supratentorial glioma, and studies with a patient cohort with less than 20 cases were excluded from the current analysis. Literature screening and selection were divided into two stages. First, investigators conducted the literature search according to the above search strategy. Second, the selected studies were validated by reviewing the abstract and intensive reading of the full text. Two investigators repeated this process. Additionally, two investigators independently performed the statistical analyses of included studies. Lastly, to verify the selection of the included literature, all of the selected studies were scored independently and subsequently reviewed by two authors (Y.L. and J.G.) using the Newcastle–Ottawa Scale (NOS) [22]. Each study was scored by population, comparability, and outcome measures. The NOS scored a full 9, with ≥ 5 meeting the inclusion criteria. Controversial studies were decided after consensus by co-authors.

### Data extraction and outcome measurement

The data from the included studies were extracted by investigators (Y.L. and J.G.). Next, the extracted data were checked independently by two authors. Data extraction was performed according to DECiMAL guidelines for meta-analysis [22]. The data extracted from the included literature was divided into basic (author, publication time, study type, and assistive technology for surgery) and clinical data (e.g., population, gender ratio, location and volume of tumor, and postoperative neurological condition). System data extraction was performed with customized sheets.

This meta-analysis aimed to assess the extent of tumor resection and its relationship to postoperative neurological deficit. For the extent of tumor resection, a 100% resection rate with no obvious lesion detected was regarded as a “gross total resection” [23, 24]. Postoperative neurological deficit evaluation was based on the criteria published by De Witt Hamer et al. [24, 25]. In brief, neurological deficits were divided into early (< 3 months) and late (> 3 months) deficits after tumor resection [25]. Additionally, postoperative neurological deficits were divided into severe (muscle strength grades 1–3, aphasia, hemianopia, and vegetative state) and non-severe (other types of neurological deficits) deficits [25]. All eligible publications that aligned with these criteria were included in this work.

### Statistical analysis

All statistical analysis was performed using the meta [26] and metafor [27] packages deployed in R (version 4.0.3) and R studio (version 1.4.1106). The mean value and 95% confidence intervals (95% CI) of the primary outcome were calculated. The Mann–Whitney *U* test was used to evaluate whether the group difference was statistically significant (IBM SPSS 26). Due to the heterogeneity of the included studies, the random effect model was used for analysis. Differences with a *P* value < 0.05 were considered significant. Egger’s test was used to detect publication bias, and *T* and *P* values were used as the analysis results. A *P* value exceeding the 0.05 value indicated no publication bias.

## Results

### Overview of the included studies

A total of 1744 relevant articles were obtained from PubMed and Embase databases (Appendix 3). Duplicated studies were deleted. After reading through the full text and re-screening, 14 papers were retained. Of those 14 studies, 1 was an RCT, and the remaining studies were observational. All of the studies were published from 2003 to 2020.

### Research characteristics and quality evaluation

Eight ISM and 6 DTI studies were incorporated into the current analysis. From these studies, a total of 1837 patients underwent surgical resection, ranging from 20 to 702 cases in each study. Included patients had an average age of 48.5 years, and 56.7% were male. All patients were diagnosed with glioma by histopathology. Regarding assistive technology, 389 patients were divided into the DTI group, and the other 1448 patients were placed into the ISM group. Because there were no included studies have clear compared of the scope of tumor resection in high-grade and low-grade glioma, it is hard to conduct subgroup analysis about extent of resection in different tumor grades. No studies which assessed the combination of the two techniques were included (summary information of included studies were shown in Table 1).

**Table 1:** Characteristics of included studies

Study	Country/ Publication time	No. of cases	DTI/ ISM	Study design	Data indicator	Quality assessment
Angel Aibar-Duran J [28]	Spain/2020	20	DTI	Cohort	abcd	High-quality
Bello L [36]	Italy/2007	88	ISM	Case series	abcd	Medium-quality
Castellano A [40]	Italy/2012	73	DTI	Case series	b	High-quality
Mo Cho J [29]	Korea/2014	103	DTI	Case series	abc	High-quality
D'Andrea G [30]	Italy/2016	27	DTI	Case series	abd	High-quality
Duffau H [31]	France/2003	103	ISM	Case series	abcd	Medium-quality
Duffau H [32]	France/2008	115	ISM	Case series	ac	High-quality
Han SJ [37]	America/2018	702	ISM	Case series	bcd	High-quality
Ius T [38]	Italy/2012	73	ISM	Case series	bc	High-quality
Javadi SA [17]	Germany/2017	20	ISM	Case series	abcd	High-quality
Evren KG [39]	America/2004	294	ISM	Case series	bc	High-quality
Ohue S [33]	Japan/2015	49	DTI	Case series	abd	High-quality
Raabe A [34]	Switzerland/2014	53	ISM	Case series	abcd	High-quality
Wu JS [35]	China/2007	118	DTI	RCT	abd	High-quality

*DTI*, Diffusion tensor imaging; *ISM*, Intraoperative subcortical mapping; *Cohort*, Cohort studies; *Case series*, Observational case series studies; *RCT*, Randomized controlled trials; *a*, Percentage of gross total resection (% GTR); *b*, Percentage of postop-early neurological deficits; *c*, Percentage of postop-late neurological deficits; *d*, Percentage of postop-severe neurological deficits

### Comparison of GTR ratio

**Fig. 1:**
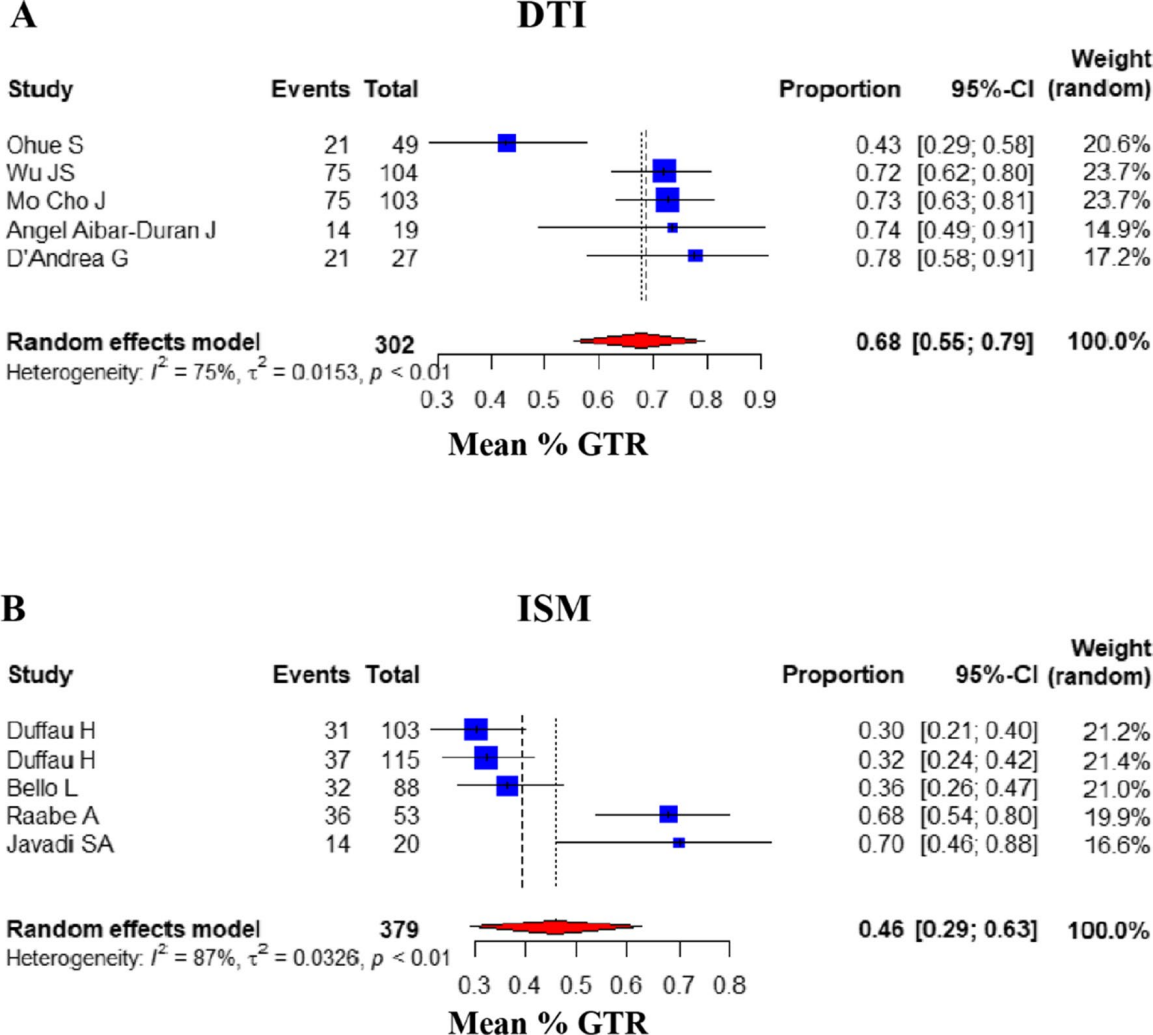
Comparison of GTR. Forest plot for pooled comparison of GTR between DTI and ISM group (random effect model). **A** DTI-navigated group vs. **B** ISM-assisted group. Comparison of GTR reveals that DTI group has higher ratio of GTR ratio, and the contrast between groups has reach statistically significant difference. Estimates of relative risk are presented by blue boxes, the area of which is proportional to the number of cases in each study. Ninety-five percent confidence intervals are indicated by a red diamond and horizontal lines

Of the 14 articles assessed, 10 reported the percentage of GTR of glioma (DTI group: 5, ISM group: 5) [7, 17, 28-35]. In this study, the mean percentage of GTR between DTI and ISM groups was assessed, and the 95% confidence intervals were calculated. The overall mean percentage of GTR for patients undergoing DTI-assisted surgery was 67.88% [95% CI: (0.5526, 0.7936)], compared with those of the ISM group (45.73%) [95% CI: (0.2914, 0.6282)]. The result of Mann–Whitney *U* test showed *P* value was 0.032. In consequence, the results of data analysis showed that the DTI group had a significantly higher GTR rate than the ISM group (Fig. 1).

### Comparison of postoperative neurological deficits

#### Postoperative early deficits

**Fig. 2:**
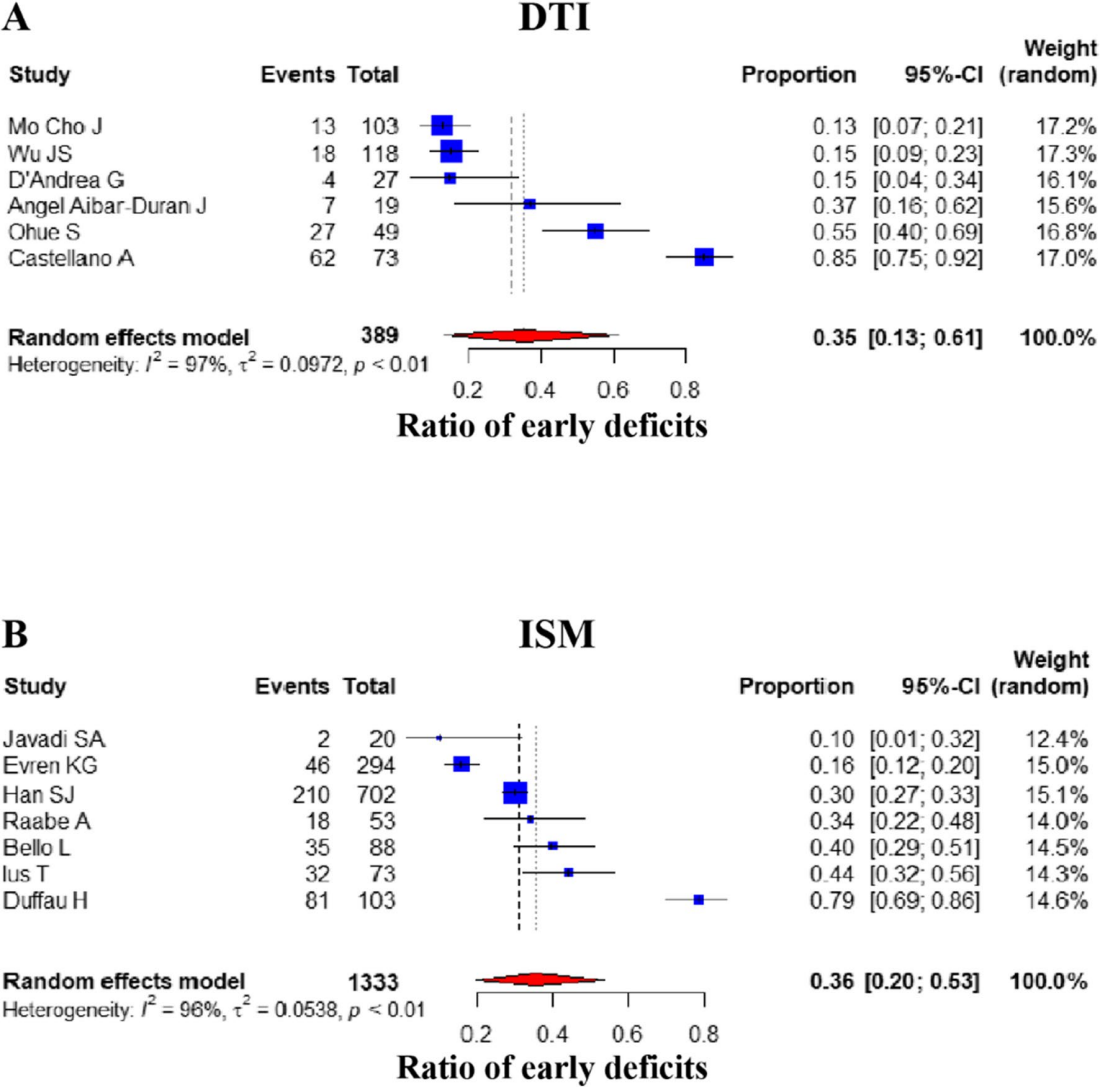
Comparison of early postoperative deficits. Forest plot for pooled comparison of postoperative early deficits between DTI and ISM group. **A** DTI-navigated group vs. **B** ISM-assisted group. Comparison of early postoperative deficits reveals that DTI group has little difference that of ISM group, the contrast between groups has not reach statistically significant difference. Estimates of relative risk are presented by blue boxes, the area of which is proportional to the number of cases in each study. Ninety-five percent confidence intervals are indicated by a red diamond and horizontal lines

The occurrence of early postoperative neurological deficits was assessed and compared proportionally in 13 studies (DTI group: 6, ISM group: 7) [17, 28-31, 33-40]. The overall random effect pooled prevalence in the DTI group (35.45%) [95% CI: (0.1325, 0.6142)] was similar to the ISM group (35.60%) [95% CI: (0.1958, 0.5342)] (*P* value = 1.000, Mann–Whitney *U* test) (Fig. 2).

#### Postoperative late deficits

**Fig. 3:**
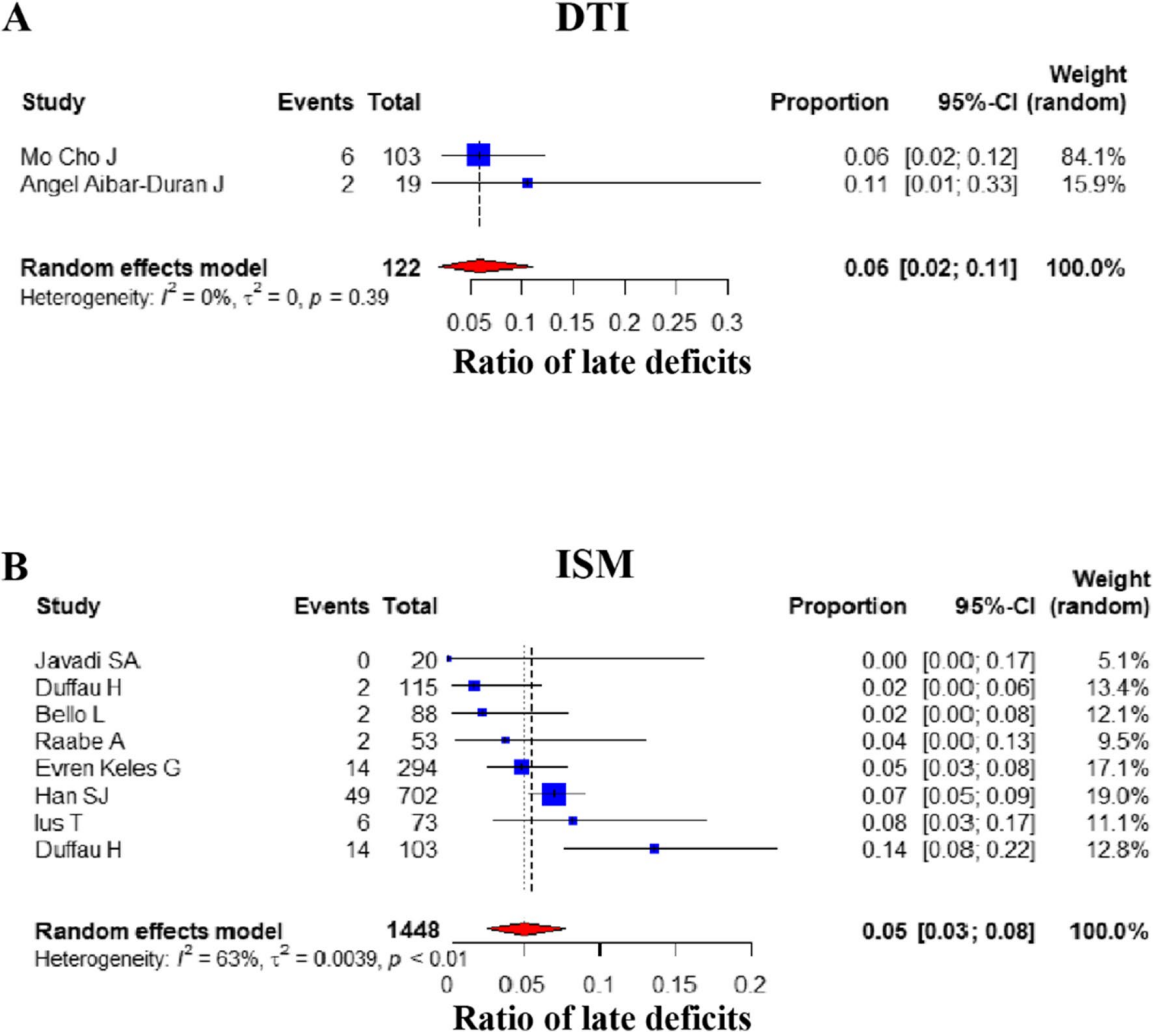
Comparison of late postoperative deficits. Forest plot for pooled comparison of postoperative late deficits between DTI and ISM group. **A** DTI-navigated group vs. **B** ISM-assisted group. Comparison of late postoperative deficits reveals that DTI group has slightly lower ratio of late postoperative deficits, and there is no statistically significant difference between groups. Estimates of relative risk are presented by blue boxes, the area of which is proportional to the number of cases in each study. Ninety-five percent confidence intervals are indicated by a red diamond and horizontal lines

Next, the occurrence of late postoperative deficits was assessed and compared proportionally in 10 studies (DTI group: 2, ISM group: 8) [17, 28, 29, 31, 32, 34, 36-39]. The overall random-effects pooled prevalence of late postoperative deficits were similar between the DTI (6.00%) [95% CI: (0.02, 0.11)] and the ISM groups (4.91%) [95% CI: (0.0262, 0.0778)] (*P* value = 1.000, Mann–Whitney *U* test) (Fig. 3).

#### Postoperative severe deficits

**Fig. 4:**
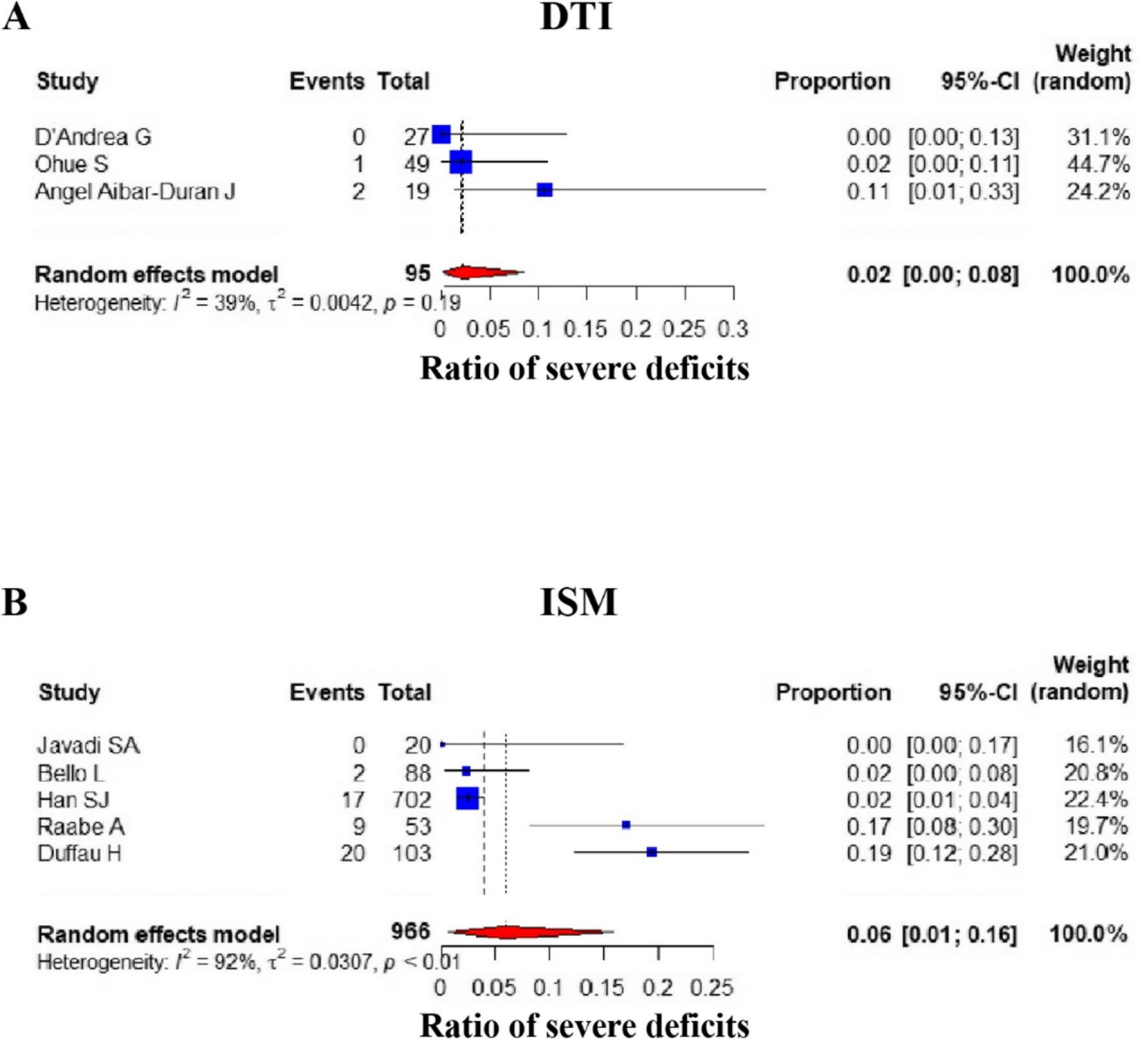
Comparison of severe postoperative deficits. Forest plot for pooled comparison of postoperative severe deficits between DTI and ISM group. **A** DTI-navigated group vs. **B** ISM-assisted group. Comparison of severe postoperative effects reveals that DTI group has lower ratio of severe postoperative deficits, and there is no statistically significant difference between groups. Estimates of relative risk are presented by blue boxes, the area of which is proportional to the number of cases in each study. Ninety-five percent confidence intervals are indicated by a red diamond and horizontal lines

Of the 14 studies assessed, severe postoperative neurological deficits, as defined by the criteria mentioned above, were identified in 8 separate studies (DTI group: 3, ISM group: 5) [17, 28, 30, 31, 33, 34, 36, 37]. The frequency of patients with severe postoperative neurological deficits was 2.21% [95%CI: (0, 0.0845)] and 5.93% [95% CI: (0.0068, 0.1585)] in the DTI and ISM groups, respectively. In summary, for deficit severity, the proportion of the DTI group was slightly lower than that of the ISM group; however, this difference was not statistically significant (*P* value = 0.393, Mann–Whitney *U* test) (Fig. 4).

### Survival analysis

A total of 5 studies (4 for the DTI group and 1 for the ISM group) documented patient survival [28, 30, 33, 35, 38]. However, it is difficult to systematically compare survival as various survival indicators (including median survival time, progression-free survival, overall survival, and 1/3/5/8-year overall survival rate.) were used in different studies (Appendix 4).

### Publication bias detection

Data on late postoperative neurological deficits in the DTI group may be affected by publication bias due to the low number of included publications. As shown in the table of Appendix 5, the *P* values showed comparison results in other groups were greater than 0.05, there were no publication bias.

## Discussion

In the current study, a systematic review and meta-analysis of relevant literature was conducted to compare surgical outcomes in glioma resection assisted by DTI or ISM. Interestingly, DTI enabled a higher rate of GTR than the ISM, but no statistical difference was found between the two groups in the functional outcome analysis. Previous studies showed that ISM could better localize subcortical fiber relative to DTI-assisted neuronavigation [7, 16, 41, 42]. These lines of evidence suggest that ISM could help surgeons better delineate the surgical limit and achieve maximal resection. However, in our meta-analysis, the DTI group achieved a higher ratio of GTR [17, 43, 44]. In general, surgeons prefer DTI to detect potential contact between fiber tracts and tumors in almost all supratentorial lesions to determine the need for subcortical mapping. As a result, gliomas in the DTI group likely had a greater mean distance to adjacent fiber tracts, resulting in a higher mean GTR rate. Moreover, all resections aided by ISM continued until eloquent pathways were identified around the surgical cavity to preserve intratumoral functional components. This approach may have contributed to a relatively lower rate (32% and 30.1%) of GTR [31, 32].

Because most studies did not strictly limit the tumor’s location, some operations were possibly performed under general anesthesia. As a result, surgical removal would mainly rely on the clinical experience of neurosurgeons rather than a subjective assessment [35, 42]. Additionally, various institutions, study duration, and assistive surgical techniques might also be responsible for the percentage of GTR difference. Even so, the advent of DTI made functional protection without awake anesthesia a reality.

Due to the limited sample size of each study, calculating the extent of resection (EOR) was challenging. In this meta-analysis, multiple studies were included, and EOR was measured at separate levels, namely gross total resection, subtotal resection, and partial resection. Consequently, the extent of lesion resection was quantified as the percentage of gross total resection (GTR) [17, 28-36]. Only one of the 14 studies calculated the specific EOR [30]. With the assistance of DTI neuronavigation, the study showed that GTR (residual = 0 cm^3^) was achieved in 20 of 27 patients. Additionally, the tumor resection rate varied from 89 to 94% in 6 patients and was unclear in the remaining patient. Each study only indicated the approximate area of the tumor location, including the frontal, parietal, and temporal lobes. Furthermore, most of the studies did not indicate whether lesions were in eloquent areas. And none of the studies conducted so far have performed a systematic analysis to compare the neurological status after surgery for different tumor grades. Due to these confounding factors, it was hard to accurately assess the difference between varying eloquent areas and tumor grades, an apt focus for future research.

Functional preservation plays a critical role in the resection of gliomas. Although DTI can identify subcortical tracts using advanced algorithms and biomathematical models, it only provides anatomical information, not functional data. The gold standard for providing functional data when operating near eloquent tracts is ISM [16, 42, 45, 46]. Previous studies have shown that ISM allows neurosurgeons to precisely map and localize subcortical white matter by applying electrical stimulation signals [16, 42, 45, 46]. Furthermore, with inhibitory and excitatory stimulation signals, neurosurgeons can observe the functional response in the surgical process [47-50]. Theoretically, ISM should provide a superior functional prognosis relative to DTI. To validate this hypothesis, we thoroughly analyzed the prevalence of functional outcomes, including early and late neurological impairment and their severity in patients treated using DTI or ISM during glioma surgery. In contrast to this hypothesis, the functional prognosis in patients treated with DTI was similar to those treated using ISM without any significant result.

Glioma resection can be improved by establishing a surgical plan using navigation software (such as Brainlab®) based on DTI data and with the assistance of intraoperative corrections such as probes, intraoperative magnetic resonance imaging, intraoperative ultrasound, and other real-time techniques [17, 51]. Previous studies have reported that intraoperative brain shift and tumor decompression released from the cerebrospinal fluid can cause fiber tract movement, making it harder for neurosurgeons to distinguish the boundary and allow for efficient resection [17]. In daily neurological routine, DTI was commonly used to rebuild a straight and robust subcortical pathway like cortical spinal tracts rather than slender and curved fibers, such as those observed in the optic tract or arcuate fasciculus. Apart from that, the complexity of the central nerve system inhibited the identification of fibers facilitating language function. Therefore, DTI only offered a limited amount of information due to the inherent constraints of the algorithm. The current study included patients with glioma in speech, motor, and optic radiation areas [17, 31, 32, 34, 36-39]. It is possible that numerous cases of glioma located in the motor area were involved in the DTI group, which did not occur in the ISM group. In tumor surgery, subcortical stimulation was necessary for motor or language mapping. With rapid advances in sequence design and image post-processing techniques, cross-fiber identification has become possible, and the automated generation of fiber tracts can be enriched without the need to set up regions of interest. Previously, cerebral edema severely affected the tracking of surrounding fiber tracts; however, this issue is gradually being resolved. Structural connectivity based on diffusion data presents global information or potential neural plasticity beyond functional details. While ISM can help neurosurgeons avoid eloquent areas during surgery, it can also lead to postoperative neurological deficits, such as seizures [43, 45]. In addition, ISM is often performed with awake craniotomy and functional tasks. A recent review indicates that awake craniotomy requires higher basic conditions of patients and may cause more postoperative neurological dysfunction (e.g., stress, anxiety, depression) [52]. Different definitions of neurological status result in a higher heterogeneity of data, potentially confounding the ability to compare neurological statuses [25]. While some patients experienced postoperative early neurological deficits, their long-term neurological outcomes were generally satisfactory. In the case of gliomas located in non-eloquent areas, ISM was not deemed necessary, and its advantages were less evident.

Whether DTI can replace ISM in glioma surgery has been a contentious and much-debated topic. Combined with our clinical diagnosis, treatment and surgical experience, we recommend using DTI independently only under the following conditions: (1) in patients with high-grade gliomas with significant contrast enhancement; (2) in patients with severe preoperative neurological deficits; (3) in cases where the lesions are located further from subcortical white matter in eloquent areas; (4) and in cases where DTI neuronavigation is better suited for stereotactic biopsy of lesions, such as those in deep areas.

The combined application of DTI and ISM can assist surgeons in preoperative assessment of surgical planning and intraoperative real-time functional status evaluation. Additionally, a combination approach uses the advantages of the two techniques and is complementary to each method’s disadvantages. Indeed, some researchers have combined DTI and ISM in the resection of glioma and observed improved patient outcomes [8, 16, 18, 42, 51]. For example, in studies conducted by Ostrý S et al. [18] and Zhu FP et al. [42], patients achieved a higher rate of GTR (68% and 69%) with a combinational approach. These studies suggest that applying DTI and ISM can enhance surgical performance and safety, assisting surgeons in achieving an efficient volumetric glioma resection. We believe that the combined application of ISM and DTI can correct brain shifts in real-time and assist with observing neurological function in patients with particular conditions (e.g., eloquent areas and fiber tract infiltration).

## Limitations

The present study had several limitations. Firstly, heterogeneity factors, such as the small number of investigations included and the lack of high-quality RCTs, may affect the results and conclusions. Most of the included studies were observational and retrospective. Hence, conclusions drawn from this meta-analysis must be interpreted with this in mind. Second, differences in technology and equipment over the 22 years (2000–2022) in which the studies were selected might bias the accuracy of the statistics. Third, a lack of preoperative neurological status might lead to a short analysis of neurological function in the perioperative period. Fourth, the lack of a subgroup analysis for low- and high-grade gliomas may result in more heterogeneity in the analysis results. Finally, many of the included studies did not accurately identify the location of the glioma, which might reduce the value of the findings.

## Conclusion

Gliomas can be safely resected using either ISM or DTI. This meta-analysis showed that both techniques could be used independently in the surgical removal of glioma. Compared with the ISM group, the DTI group achieved a higher rate of GTR. The postoperative neurological function between groups was similar. These data suggest that DTI is a satisfactory substitute for ISM in glioma surgery. Moreover, the combination of ISM and DTI is theoretically superior to using either method alone.

### Supplementary Information

Below is the link to the electronic supplementary material.

Supplementary file1 (DOCX 66 KB)

Supplementary file2 (DOCX 35 KB)

## Data Availability

The datasets generated during and analyzed during the current study are available from the corresponding author on reasonable request.

## Abbreviations

DTI: Diffusion tensor imaging
ISM: Intraoperative subcortical mapping
EOR: Extent of resection
GTR: Gross total resection
NOS: Newcastle-Ottawa Scale
RCT: Randomized controlled trials
