# Supplementary material for: Diffusion tensor imaging versus intraoperative subcortical mapping for glioma resection: a systematic review and meta-analysis
Source: Neurosurg Rev. 2023 Jun 28;46(1):154. doi: 10.1007/s10143-023-02058-5 (PMC10307847; doi:10.1007/s10143-023-02058-5)
Supplement: Supplementary file 1 — Supplementary file1 (DOCX 66 KB) [file 10143_2023_2058_MOESM1_ESM.docx]

**Title:**

Diffusion tensor imaging versus intraoperative subcortical mapping for glioma resection: a systematic review and meta-analysis

**Journal:**

Neurosurgical Review

**Authors:**

Yiming Li, M.D.,^1,^* Jiahe Guo, M.D.,^1,^* Kai Zhang, M.D.,^2,3^ Huijie Wei, M.D.,^1^ Jikang Fan, M.D.,^1^ Shengping Yu, M.D.,^1^ Tao Li, M.D.,^1^ Xuejun Yang, M.D^3,4^

**Affiliations:**

1 Department of Neurosurgery, Tianjin Medical University General Hospital, Tianjin, China

2 Department of Surgery, First Teaching Hospital of Tianjin University of Traditional Chinese Medicine, Tianjin, China

3 Department of Neurosurgery, Tsinghua University Beijing Tsinghua Changgung Hospital, Beijing, China

4 Institute for Intelligent Healthcare, Tsinghua University, Beijing, China

*These authors contributed equally to this work

**Corresponding Author’s name and current institution**

Tao Li, M.D., Ph.D

Department of Neurosurgery, Tianjin Medical University General Hospital, Tianjin, China

Xuejun Yang, M.D., Ph.D, ORCID: 0000-0001-7056-1223

Department of Neurosurgery, Tsinghua University Beijing Tsinghua Changgung Hospital, Beijing, China; Institute for Intelligent Healthcare, Tsinghua University, Beijing, China

**Corresponding Author’s Email:** [litao@tmu.edu.cn](mailto:litao@tmu.edu.cn), [ydenny@126.com](mailto:ydenny@126.com)

**Appendix 1. Search Strategy in PubMed**

(((("Diffusion Tensor Imaging"[Mesh]) OR ((((((((Imaging, Diffusion Tensor) OR (Diffusion Tensor Magnetic Resonance Imaging)) OR (Diffusion Tensor MRI)) OR (Diffusion Tensor MRIs)) OR (MRI, Diffusion Tensor)) OR (DTI MRI)) OR (Diffusion Tractography)) OR (Tractography, Diffusion))) OR ((((((((((((((((((((((((((((((((((((Continuous subcortical stimulation) OR (Direct electrical subcortical stimulation)) OR (Direct stimulation mapping of subcortical portions)) OR (Intraoperative cortical-subcortical stimulation and cortical mapping)) OR (Intraoperative cortico-subcortical mapping monitoring)) OR (Intraoperative subcortical direct electrostimulation)) OR (Intraoperative direct subcortical stimulation)) OR (Intraoperative electrical mapping of subcortical portions)) OR (Intraoperative electrical subcortical mapping)) OR (Intraoperative electrostimulation mapping)) OR (Intraoperative mapping)) OR (Intraoperative motor/language mapping)) OR (Intraoperative subcortical electrical mapping)) OR (Intraoperative subcortical electrical stimulation/electrostimulation)) OR (Intra-operative subcortical electrical stimulation)) OR (Intraoperative subcortical electrical stimulations)) OR (Intraoperative subcortical electrostimulation mapping)) OR (Intraoperative subcortical electrostimulation)) OR (Intraoperative subcortical fiber mapping)) OR (Intraoperative subcortical functional brain mapping)) OR (Intraoperative subcortical functional mapping)) OR (Intraoperative subcortical mapping)) OR (Intraoperative subcortical motor evoked potential stimulation)) OR (Intraoperative subcortical stimulation mapping)) OR (Intraoperative subcortical stimulation)) OR (Intraoperative subcortical stimulations)) OR (Subcortical cerebral mapping)) OR (Subcortical dynamic mapping)) OR (Subcortical electrical intraoperative mapping)) OR (Subcortical electrical mapping)) OR (Subcortical electrical stimulation mapping)) OR (Subcortical electrical stimulation)) OR (Subcortical electrostimulation mapping)) OR (Subcortical electrostimulation)) OR (Subcortical mapping)) OR (Subcortical stimulation mapping))) AND ((((Brain Neoplasms[mesh]) OR (Supratentorial Neoplasms[mesh])) OR (Glioma[mesh])) AND (adults[mesh]))) NOT ((((((((((((((((case reports[pt]) OR (comment[pt])) OR (editorial[pt])) OR (technical report[pt])) OR (infratentorial neoplasms[majr])) OR (brain stem neoplasms[majr])) OR (cerebellar neoplasms[majr])) OR (cerebral ventricle neoplasms[majr])) OR (pinealoma[majr])) OR (hypothalamic neoplasms[majr])) OR (pituitary neoplasms[majr])) OR (skull base neoplasms[majr])) OR (subarachnoid hemorrhage[majr])) OR (intracranial aneurysm[majr])) OR (brain abscess[majr])) OR (neurocutaneous syndromes[majr])) AND (2000:2022[pdat])

**Appendix 2. Search Strategy in Embase**

('diffusion tensor imaging'/exp OR 'diffusion tensor imaging' OR 'subcortical stimulation mapping') AND ('glioma'/exp OR glioma) AND ('adult'/exp OR adult) NOT ('case report'/exp OR 'case report') NOT comment NOT ('editorial'/exp OR editorial) NOT ('technical report'/exp OR 'technical report') NOT ('cerebellum tumor'/exp OR 'cerebellum tumor') NOT ('brain stem tumor'/exp OR 'brain stem tumor') NOT ('infratentorial neoplasms'/exp OR 'infratentorial neoplasms') NOT ('brain ventricle tumor'/exp OR 'brain ventricle tumor') NOT ('pineal body tumor'/exp OR 'pineal body tumor') NOT ('hypothalamus tumor'/exp OR 'hypothalamus tumor') NOT ('hypophysis tumor'/exp OR 'hypophysis tumor') NOT ('skull base tumor'/exp OR 'skull base tumor') NOT ('subarachnoid hemorrhage'/exp OR 'subarachnoid hemorrhage') NOT ('intracranial aneurysm'/exp OR 'intracranial aneurysm') NOT ('brain abscess'/exp OR 'brain abscess') NOT ('phakomatosis'/exp OR phakomatosis)

**Appendix 3. Research retrieval flow chart.**

**Identification of studies via other methods**

**Identification of studies via databases and registers**

Total of 1744 articles were retrieved:

PubMed (n = 1004)

Embase (n = 740)

Records identified from:

Citation supplement (n = 10)

**Initial Search**

Literatures removed *before screening*:

Duplicate records removed (n = 533)

Selection after duplicated articles excluded (n = 1211)

Literatures excluded for non-conforming literature (n = 1115)

Selection after title and abstract reading (n = 96)

**Rigorous Selection**

Reports excluded:

Reason 1: Number of cases below 20 (n = 20)

Reason 2: Cortical or subcortical stimulation mapping is not clear (n = 30)

Reason 3: Poor data quality or incomplete data (n = 33)

Reason 4: Study shared data (n = 1)

Reports excluded:

Reason 1: Number of cases below 20 (n = 3)

Reason 2: Cortical or subcortical stimulation mapping is not clear (n = 2)

Reason 3: Poor data quality or incomplete data (n = 3)

Selection after full text review

(n = 12)

Literatures assessed for eligibility

(n = 2)

Publications included for analysis

(n = 14)

**Publication Selection**

After an initial search and rigorous selection, 14 studies were selected for subsequent meta-analysis

**Appendix 4. Survival analysis**

| Study | DTI/ISM | Survival indicator |
| --- | --- | --- |
| Wu JS[1] | DTI | MST: 21.2 months (95% CI: 14.1-28.3 months) |
| Ohue S[2] | DTI | PFS: 9.7 months, OS: 16.3 months |
| D’Andrea G[3] | DTI | OS: 16.55 months |
| Aibar-Durán JÁ[4] | DTI | 1-, 3- and 5 - year OS rate: 94.7%, 47.3% and 42.1% |
| Ius T[5] | ISM | 5-year OS rate: 80%; 8-year OS rate: 66% |

**Abbreviations:** MST: Median survival time, PFS: Progression free survival, OS: Overall survival.

**Appendix 5. Analysis of publication bias**

| Data indicator | DTI/ISM | | t | df | *p*-value |
| --- | --- | --- | --- | --- | --- |
| % GTR | DTI | 1.13 | | 2 | 0.3745 |
|  | ISM | 0.5 | | 1 | 0.7049 |
| Postop-early deficits | DTI | 0.49 | | 4 | 0.6514 |
|  | ISM | 0.7 | | 5 | 0.5061 |
| Postop-late deficits | DTI | — | | | |
|  | ISM | -0.75 | | 6 | 0.4830 |
| Postop-severe deficits | DTI | -0.51 | | 2 | 0.6615 |
|  | ISM | 0.79 | | 3 | 0.4880 |

**Abbreviations:** % GTR: Percentage of gross total resection; Postop-early deficits: Postoperative early neurological deficits; Postop-late deficits: Postoperative late neurological deficits; Postop-severe deficits: Postoperative severe neurological deficits; DTI: Diffusion tensor imaging, ISM: Intraoperative subcortical mapping.

**Appendix Reference**

1. Wu, J.S., et al., *Clinical evaluation and follow-up outcome of diffusion tensor imaging-based functional neuronavigation: a prospective, controlled study in patients with gliomas involving pyramidal tracts.* Neurosurgery, 2007. **61**(5): p. 935-948; discussion 948-9.

2. Ohue, S., et al., *Surgical results of tumor resection using tractography-integrated navigation-guided fence-post catheter techniques and motor-evoked potentials for preservation of motor function in patients with glioblastomas near the pyramidal tracts.* Neurosurg Rev, 2015. **38**(2): p. 293-306; discussion 306-7.

3. D'Andrea, G., et al., *Safe Resection of Gliomas of the Dominant Angular Gyrus Availing of Preoperative FMRI and Intraoperative DTI: Preliminary Series and Surgical Technique.* World Neurosurg, 2016. **87**: p. 627-639.

4. Aibar-Durán, J., et al., *Intraoperative Use and Benefits of Tractography in Awake Surgery Patients.* World Neurosurg, 2020. **137**: p. e347-e353.

5. Ius, T., et al., *Low-grade glioma surgery in eloquent areas: volumetric analysis of extent of resection and its impact on overall survival. A single-institution experience in 190 patients: clinical article.* J Neurosurg, 2012. **117**(6): p. 1039-1052.
